# Enhancement of Efficacy of Retinoids through Enhancing Retinoid-Induced RAR Activity and Inhibiting Hydroxylation of Retinoic Acid, and Its Clinical Efficacy on Photo-Aging

**DOI:** 10.3390/pharmaceutics14112412

**Published:** 2022-11-08

**Authors:** Seongsu Kang, Hyejin Lee, Seung-Hyun Jun, Sun-Gyoo Park, Nae-Gyu Kang

**Affiliations:** LG Household and Health Care R&D Center, Seoul 07795, Korea

**Keywords:** retinol, retinoid, cosmetics, photoaging, retinoic acid receptor, hydroxylation

## Abstract

Retinoids, one of the most robust bioactive materials, have been widely used to improve various dermatological and pathological conditions. The body has an endogenous mechanism that modulates the exogenous retinoid above physiological concentrations, which limits the bioavailability or pharmacological efficacy of retinoids. Considering that most retinoids trigger extensive irritation in users, it is necessary to enhance the pharmacological efficacy of retinoids, thereby achieving a higher efficacy at a lower dosage. Here, we present approaches for enhancing the efficacy of retinol by enhancing retinoid-induced RAR gamma (RAR-γ) activity and inhibiting the hydroxylation of retinoic acid. Using both in vitro and ex vivo experiments, retinoid boosters were demonstrated to enhance pharmacological efficacy. A small pilot study was conducted to investigate the efficacy for improvement of facial wrinkles, whose results revealed that these boosters could enhance the pharmacological efficacy of topical applications of both retinol and retinoic acid for cosmetic use. These results promote not only a higher compliance among retinoids users, but also provide significant insights into the mechanisms underlying the action of retinoids.

## 1. Introduction

Throughout human history, from ancient Egypt, when individuals recognized the relationships between night blindness and vitamin A deficiency, to more recent times, retinoids, a class of compounds derived from vitamin A, have been extensively used and studied, with diverse biomedical applications. The primary retinoid, retinoic acid (RA), has been utilized for the treatment of cancer, ocular disease, neurodegenerative disease, and skin disorders, which are all mediated by interactions with nuclear receptors [1].

The predominant clinical application of retinoids is its dermatological use via both oral and topical applications. The therapeutic efficacy of retinoids on skin diseases, including psoriasis, ichthyosis, actinic keratosis, acne, and fungal infections have been consistently reported [2,3,4,5,6]. Similarly, its therapeutic efficacy on photoaging has also investigated, and the topical application of retinoids is regarded as the first-line remedy for photoaging, subject to the US Federal Drug Administration’s (FDA) approval in 1995. Extensive clinical studies and practical applications of tretinoin (all-trans retinoic acid, RA) in the treatment of photoaging has inspired the development of cosmetic-level retinoids, such as retinol, retinyl palmitate, and retinaldehyde. Since these, research has revealed that cosmetic retinoids are also effective for the treatment of photoaging, improving fine and coarse wrinkles, skin roughness, and abnormal pigmentation [7]. Retinoids are believed to exert its beneficial effects on the skin through an increase in epidermal thickness, the stimulation of collagen synthesis, and the reduced expression of matrix metalloproteinase, which could normalize aged skin tissue [8].

However, the bioavailability and pharmacologic efficacy of retinoids remain limited in most cases, leading to a large variability in terms of clinical outcomes across populations. Variability in its efficacy is prominently observed in some cancerous disease models, such as acute promyelocytic leukemia (APL), which is characterized by truncated retinoic acid receptor alpha (RAR-α) [9]. In APL, for which RA is used as the treatment, RA-therapy resistance is frequently observed. This leads reduced a therapeutic efficacy and even disease relapse. Previous studies have shown that mutations in the ligand binding domain (LBD) of the RAR-α region of PML-RARα reduce RA binding, which suggested that defective RA binding is a major cause of RA resistance [10,11]. These observations imply that RAR binding or interaction with retinoids critically affect the efficacy of retinoids.

Furthermore, potential negative feedbacks resulting from the modulation of exogenous retinoid levels above physiological concentrations should also be considered. It has been shown that most tissues possess a mechanism to regulate retinoid levels, and that cytochromes P450 (CYPs) metabolize intrinsic or endogenous retinoid levels for homeostasis [12]. In the case of APL, as mentioned above, studies have pointed out a significant relationship between retinoic acid sensitivity and CYP26 expression levels [13]. Additionally, research has found that APL relapse following RA treatment may be mediated by increased CYP26 expression during treatment, which could be prevented by CYP26 inhibition [14,15]. Similarly, with regards to other types of cancer, their retinoic acid resistance has been found to be highly related to elevated CYP26 levels [16], wherein treatment with retinoic acid metabolism blocking agents (RAMBA) could increase the concentration of retinoic acid and compensate retinoic acid resistance by increasing the retinoic acid potency, as proved by preclinical and early clinical data [14,15].

Considering the inherent variability of retinoid bioavailability and the innate feedback mechanism for exogenous retinoid, the enhancement of retinoid efficacy is necessary to improve clinical outcomes for cosmetic use of retinoids. Additionally, when taking account of retinoid-induced irritation, which is frequently observed after topical application [17,18], a higher efficacy at a lower dosage is ultimately desired. In this study, we propose a comprehensive strategy with which to enhance topically applied retinoids for a cosmetic use by: (1) enhancing the activation of RAR by retinoids, and (2) inhibiting the enzymatic hydroxylation of retinoic acid in a direct or indirect manner. First, we demonstrate that camphor, a terpenoid found in the wood of the camphor laurel, enhances retinol-induced RAR-γ activation, as well as its pharmacological efficacy, both in vitro and ex vivo. For the inhibition of RA hydroxylation by keratinocyte, an azolic compound, prolinamidoethyl imidazole, and some phytochemicals, including apigenin, baicalin, and luteolin, were found to inhibit the hydroxylation of retinoic acid on keratinocytes. Furthemore, RAMBA-mimicking compounds were demonstrated to enhance the responses of retinoic acid in the skin both in vitro and in vivo.

Interestingly, further in vivo human clinical tests verified that a retinoids booster formula comprised of the compounds mentioned above markedly enhanced the clinical efficacy of retinol and retinoic acid on photoaging as a cosmetic use, with improvements observed in the appearance of wrinkles upon their application. This comprehensive topical formulation contributes not only to the cosmetic use of retinoids, but also wide range of uses for the treatment of various diseases, including acne, psoriasis, and alopecia. This research and the observations reported in this study provide a deeper understanding of the mechanism underlying the action of retinoids in the skin.

## 2. Materials and Methods

### 2.1. Cell Culture and Preparation

Fibroblasts were cultured with DMEM (Gibco, Waltham, MA, USA) supplemented with 10% FBS (Gibco, Waltham, MA, USA), penicillin-streptomycin (Gibco, Waltham, MA, USA) at 37 °C with 5% CO_2_. HaCaT were cultured with DMEM supplemented with 10% FBS, penicillin-streptomycin (Gibco, Waltham, MA, USA), 1 mM sodium pyruvate (Gibco, Waltham, MA, USA), 2 mM L-glutamine (Gibco, Waltham, MA, USA), and 0.01 mM CaCl_2_ (Sigma-Aldrich, St. Louis, MI, USA) at 37 °C with 5% CO_2_.

Camphor (1,7,7-Trimethylbicyclo [2.2.1]heptan-2-one), prolinamidoethyl imidazole, apigenin (4′,5,7-Trihydroxyflavone), baicalin (2S,3S,4S,5R,6S)-6-[(5,6-Dihydroxy-4-oxo-2-phenyl-4H-1-benzopyran-7-yl)oxy]-3,4,5-trihydroxyoxane-2-carboxylic acid), and luteolin (3′,4′,5,7-Tetrahydroxyflavone) were purchased from Sigma-Aldrich (St. Louis, MI, USA).

For ex-vivo experiment, 3D skin (Neoderm^®^, Tego Science, Seoul, Korea) was purchased from Tego Science. Maintenance and culture followed manufacturer’s instruction. In the experiment for RA hydroxylation (Section 3.2.2), retinoic acid and hydroxylation inhibitors were added in the culture media. For experiment of retinol booster (Section 3.3), retinol 3300 IU and boosters (each 0.1%) was topically applied. The 3 D skin was cultured for 7 days, and stained by hematoxylin-eosin (H&E) or Masson’s trichrome. For measurement of IL-1α, ELISA was performed through manufacturer’s instruction (Human IL-1 alpha/IL-1F1 Quantikine^®^ ELISA Kit, R&D Systems, Minneapolis, MI, USA).

For measurement of cell viability, cell counting kit-8 (CCK-8, Dojindo, Rockville, MD, USA) was performed according to manufacturer’s protocol.

### 2.2. RT-qPCR & ELISA

RNA was extracted from fibroblast and HaCaT using RNeasy mini kit (Qiagen, Hilden, Germany). Total RNA (1 μg) was reverse-transcribed into cDNA using cDNA synthesis kit (Philekorea, Seoul, Korea) following manufacturer’s protocol through Veriti 96 Well Thermal Cycler (Applied Biosystems, Waltham, MA, USA). The following temperature protocol was used for reverse transcription: 42 °C for 30 min and 72 °C for 10 min. Amplification of cDNA was performed using the StepOnePlus™ RT-PCR system (Applied Biosystems, Waltham, MA, USA) using TaqMan™ Universal PCR Master Mix (Applied Biosystems, Waltham, MA, USA) according to protocol by manufacturer. The following thermocycling conditions were used for qPCR: 30 cycles at 95 °C for 45 s, 60 °C for 1 min and 72 °C for 45 s. The total RNA (1 μg) was used as the template for cDNA synthesis and PCR using the SYBR Green PCR Master mix (TOP real™ SYBR Green qPCR High-ROX PreMIX, Enzynomics, Seoul, Korea). The following primers were used: GAPDH 5′-ACCACAGTCCATGCCATCAC-3′/5′-TCCACCACCCTGTTGCTGTA-3′; CYP26A1 5′-ATGTTCCGAATCGCCATGC-3′/5′-GAGAAGAGATTGCGGGTCATTT-3′; CYP26B1 5′-TGGACCTCCTCATTGAGAGCA-3′/5′ GGCATAGGCCGCAAAGATCA-3′; CYP26C1 5′- GCTGGAGCGTGATGTATAGCA-3′/5′-GCCGAACGGGATGTAATGGA-3′; CYP3A4 5′- CACGAGCAGTGTTCTCTCCTT-3′/CACAGTATCATAGGTGGGTGGT-3′; CYP3A5 5′-ATTGGCATGAGGTTTGCTCTC-3′/5′-AAGTCCTTGCGTGTCTAATTTCA-3′; COL1A1 5′-GATTCCCTGGACCTAAAGGTGC-3′; 5′-AGCCTCTCCATCTTTGCCAGCA-3′.

For measurement of ELISA for human pro-collagen I alpha 1 was performed through manufacturer’s instruction (Human Pro-Collagen I alpha 1 DuoSet ELISA, R&D Systems, Minneapolis, MI, USA).

### 2.3. Retinoid Metabolites Analysis Based on HPLC

Overall experimental procedure followed the previous literature in which observed that culture conditions under serum or not, and passage critically affect RA metabolism and induction of 4-hydroxylase activity [19]. Briefly, HaCaT was seeded and cultured in serum-containing medium for 48 h. Cells at confluency were treated with RA 1 µM (Sigma-Aldrich, St. Louis, MI, USA) and methanol (Sigma-Aldrich, St. Louis, MI, USA) and incubated for additional 48 h. Cultured media and cell lysate were freeze-dried and resuspended in methanol. This methanol-based solution was analyzed by HPLC (Shimadzu, SCL-40, LC-40DXR, CTO-40C etc., Kyoto, Japan). Detailed procedure followed the previous research [20]. 60 mM ammonium acetate (Sigma-Aldrich, St. Louis, MI, USA) adjusted to pH 5.75 with acetic acid (Sigma-Aldrich, St. Louis, MI, USA) and methanol (Sigma-Aldrich, St. Louis, MI, USA) were used as HPLC buffers. The flow rate was 1 mL/min. The gradient conditions were (1) 15% of methanol at the time of injection, (2) linear increase to 99% of methanol at the 30 min, and (3) maintenance of 99% of methanol for 15 min. UV detection was carried out at 340 nm.

### 2.4. RAR Activation Assay

RAR-γ reporter cell lines were purchased from BPS bioscience (San Diego, CA, USA). Cells were maintained according to the manufacturer’s guide. Briefly, the frozen cells were quickly thawed and transferred in Dulbecco’s Modified Eagle’s Medium (DMEM) supplemented with 10% FBS, 1% Penicillin/Streptomycin (P/S). At first passage, switch to complete medium (DMEM with 10% FBS, 1% P/S, 400 µg/mL of Geneticin (G418) (#11811031, Invitrogen, Waltham, MA, USA), 1 µg/mL of Puromycin (#SV30075.01, Hyclone, Logan, UT, USA), and 100 μg/mL Hygromycin (#SV30070.01, Hyclone, Logan, UT, USA). Cells were incubated at 37 °C with 5% CO_2_. Cells were seeded in a black 96-well plate and cultured overnight. Then, cells were treated with retinol, retinaldehyde, retinoic acid or camphor respectively with mentioned concentrations for 24 h. The RAR-γ activation of each condition was quantified by measuring luciferase activity using the Steady-Glo luciferase assay system (Promega, Madison, WI, USA).

### 2.5. Human Test

This study was approved by the Ethics committee of LG H&H Institutional Review Board (LGHH-20220113-AA-04-02). Prior to participation in the study, human test subjects were informed of possible side effects and consented to the study. Total twenty people whose ages ranged from 40 to 60 were tested. Ten people were assigned for a test for retinol, and others were assigned for a test for retinoic acid. A corresponding amount of retinol or retinoic acid were added to the typical O/W-type cream previously developed in our research institution. The cream formulation is composed of following components; Cetyl stearyl alcohol, stearyl alcohol, glyceryl stearate, cetearyl alcohol, PEG-40 stearate, ceteareth-20, beeswax, C14-22 alcohols, C12-20 alkyl glucoside, lecithin, tocopherol, caprylic/capric triglyceride, squalene, cyclopentasiloxane, cyclohexasiloxane, dimethicone/vinyl dimethicone crosspolymer, isocetyl myristate, dipropylene glycol, glycerin, betaine, 1,2-hexanediol, EDTA-3Na, xanthan gum, carbomer(2-propenoic acid, polymer with 2,2-bis(hydroxymethyl)propane-1,3-diol 2-propenyl ether), tromethamine, and distilled water. All types of cream used in this study was produced in the LG H&H R&D center by own protocol. For human test (Results and Discussion Section 3.3), the following booster ingredients were added; Cinnamomum Camphora leaf extract (Camphor) 1%, Silybum Marianum extract (Silymarin) 1%, Prolinamidoethyl imidazole 0.1%, Scutellaria Baicalensis Root Extract (Baicalin) 1%, and Chamomilla Recutita extract (Apigenin) 1%. The concentrations of the actives in the plant extract were managed between 150~2000 ppm.

Before measurement, the face of subjects was cleaned and acclimated for 20 min in an air-conditioned room (temperature 22 ± 2 °C; relative humidity 50 ± 10%). Wrinkle, pigmentation, and skin texture on the face was measured by Antera 3D (Miravex, Dublin, Ireland). Subjective satisfaction for improvement of skin appearance was asked after experiment.

## 3. Results and Discussion

### 3.1. Camphor Enhances Retinol-Induced RAR Activation in Fibroblasts

#### In Vitro Experiments Using Retinol and Camphor

Most retinoids are transformed to retinoic acid via numerous metabolic pathways, which ultimately induce the pharmacological effects of this compound via RAR activation. Though some studies have reported on retinoid metabolism in the skin in vitro and ex vivo, it is has yet to be empirically proven how retinoids are metabolized in the skin in vivo in terms of kinetics. In the case of retinol, an extensively used type of retinoids, two representative studies exist. One study found that topical application of retinol resulted in the detection of only 0.8% retinoic acid in the epidermis and dermis, while the other study did not detect retinoic acid, even retinaldehyde [21,22]. These findings imply that conversion of retinol into retinoic acid is not quite dominant and there is another pathway how retinol trigger biological effects.

Furthermore, it has been suggested that non-retinoic acid retinoids may trigger biological effects via RAR activation by themselves. Another study found that all-trans-retinol binds to all three RAR subtypes with a ~4–7-fold weaker potency than RA [23].

It is also worth noting that, in terms of APL, the variability inherent to RAR also affects RAR activation, and thus the efficacy of retinoic acid. In this context, enhancing RAR activation by retinoids may increase their pharmacologic efficacy. Considering that RAR-γ is main subtype among the three RAR subtypes in human skin, accounting for approximately 87% [24], in the present study, the mechanism by which RAR-γ reacts to retinoids and retinoids boosters was investigated.

**Figure 1:**
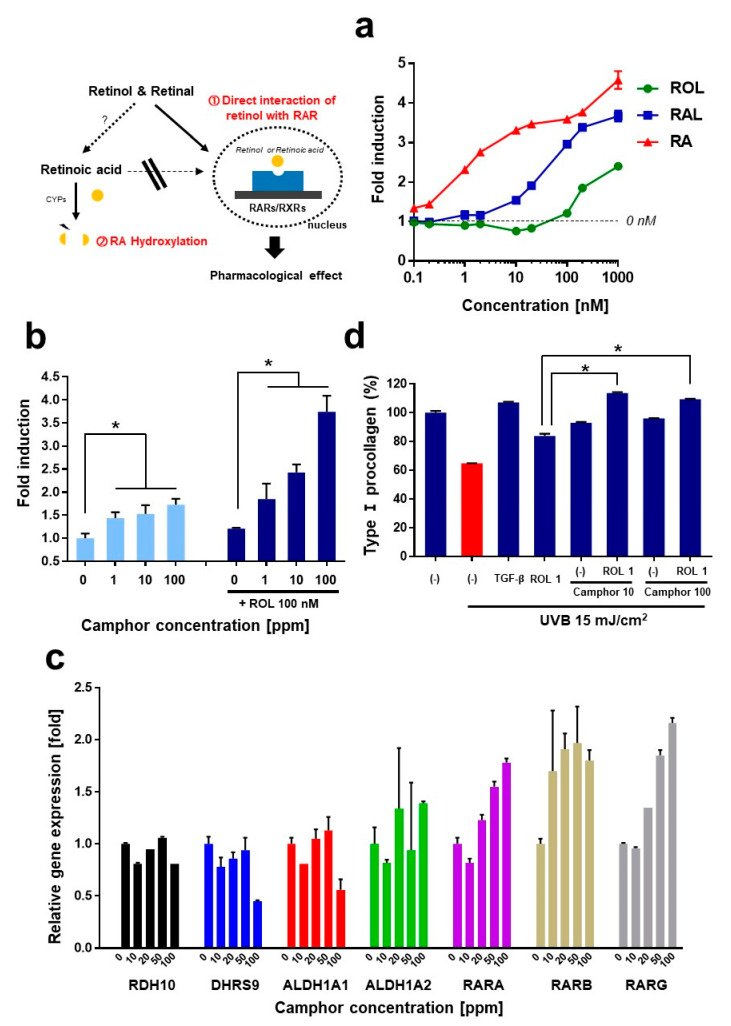
RAR-γ activation by retinoids and camphor. (**a**) RAR-γ activation by retinoids. Retinoic acid (RA), retinaldehyde (RAL), and retinol (ROL) were analyzed. RAR-γ activation in the reporter cell line was measured by luciferase activity. (**b**) RAR-γ activation by camphor. (**c**) Relative mRNA expression of retinoid metabolism-related genes. (**d**) Type-1 procollagen expression of fibroblast. * Significantly different results (Student’s *t*-test, *p* <  0.05).

First, the retinoid-induced activation of RAR-γ was investigated. As a result, retinol was found to activate RAR-γ with a lower potency than retinoic acid and retinaldehyde (Figure 1a). This significant difference in RAR-γ activation seems to be one of the causes for the weaker effects of retinol on the appearance of wrinkles compared to retinoic acid. In the present study, we attempted to identify a synergistic substance that could increase the activation of RAR-γ by retinol. As a result, camphor was found to activate RAR-γ, as well as boost retinol-induced RAR-γ activation (Figure 1b). In this respect, camphor was found to significantly enhance the retinol-induced activation of RAR-γ (1.21-fold), showing a 1.85-, 2.43-, and 3.74-fold increase upon treatment with 1, 10, and 100 ppm of camphor, respectively. No significant cytotoxicity was observed after camphor treatment (Supplementary Figure S1).

Next, we investigated how camphor modulates the expression of genes considered to be involved in retinoid metabolism (Figure 1c). Interestingly, camphor was found to primarily induce the overexpression of three retinoid receptor genes, RARA, RARB, and RARG. After treatment with 100 ppm of camphor, the expression levels of RARA, RARB, and RARG was found to increase by 1.78-, 1.9-, and 2.16-fold, respectively. In the case of RDH10, DHRS9, ALDH1A1, and ALDH1A2, which are enzymes involved in the conversion of retinol to retinoic acid, no increase in gene expression was observed during camphor treatment at any concentration.

Thereafter, whether camphor enhances the pharmacological efficacy of retinol was investigated in both in vitro and ex vivo models. In the in vitro experiments, camphor was found to enhance the effect of retinol on mitigating the UVB-induced decrease of collagen expression (Figure 1d). Upon treatment with 10 and 100 ppm of camphor, the expression of collagen was found to increase by 22.25% (93% to 113.7%) and 13.85% (96% to 109.3%) respectively. Interestingly, camphor itself was found to induced type-I collagen in UVB-treated fibroblasts, showing a 93% and 96% increase after treatment with 10 and 100 ppm of camphor compared to negative control, respectively. The experimental results for RAR-γ activation, as well as the expression patterns for retinoids metabolism-related genes, suggest that camphor acts as a retinol.

### 3.2. Enhancement of Retinoids Efficacy by Inhibition of RA Hydroxylation

#### 3.2.1. Screening of Materials Inhibiting the Hydroxylation of Retinoic Acid Using a HPLC-Based Method

Previous studies have shown that retinoic acid is actively metabolized in the skin [25]. It has been reported that many types of CYP enzymes are involved in the hydroxylation of retinoic acid into 4-OH-RA, 4-oxo-RA, 18-OH-RA, and 5,6-epoxy RA. In the skin, RA is primarily catabolized to 4-hydroxy tRA. Furthemore, it has been reported that some species of CYP 450, such as CYP26A1, are robustly involved in the hydroxylation of retinoic acid [26]. Pioneering research on the hydroxylation of RA in the skin has demonstrated that CYP26A1 is only expressed at negligible levels under normal conditions, while its expression increases three-fold in keratinocytes after RA treatment [26]. Research has also found that the expression of CYP26A1 is restricted in basal keratinocytes, which is in agreement with an earlier study in which the hydroxylation of RA by HaCaT was only observed upon culturing under calcium-free conditions, which represents the proliferation stage of keratinocytes in the basal layer [19].

Previous reports have argued that RA hydroxylation seems to physiologically regulate both the endogenous and exogenous levels of RA, primarily affecting the bioavailability of exogenous retinoids. RA hydroxylation seems to initiate the degradation of RA for its ultimate elimination. Moreover, 4-hydroxy RA has been reported to bind to RARs with 100-fold less affinity and 10-fold less potency than RA [25], which implies that RA hydroxylation greatly hinders pharmacological efficacy.

Interestingly, previous studies have found that liarozole and climbazole can increase the bioavailability of topically-applied retinoids. Liarozole treatment was found to enhance cellular and molecular responses to retinol in the skin in vivo by inhibiting human epidermal retinoic acid 4-hydroxylase activity [27], while climbazole boosted the response of retinol in in vivo experiments [28]. However, most RAMBA have not been approved for practical use as drugs or cosmetics, and climbazole, which is well-known for its use as an antifungal agent for the treatment of dandruff, has been a subject to controversy due to safety issues. In fact, its use in cosmetic products was recently restricted in the European Union (European Commission (EC), May 2019 (EU 2019/698)). Another type of RAMBA for a dermatological use, ketoconazole, has also been reported to cause allergies and anaphylactic reactions [29].

**Figure 2:**
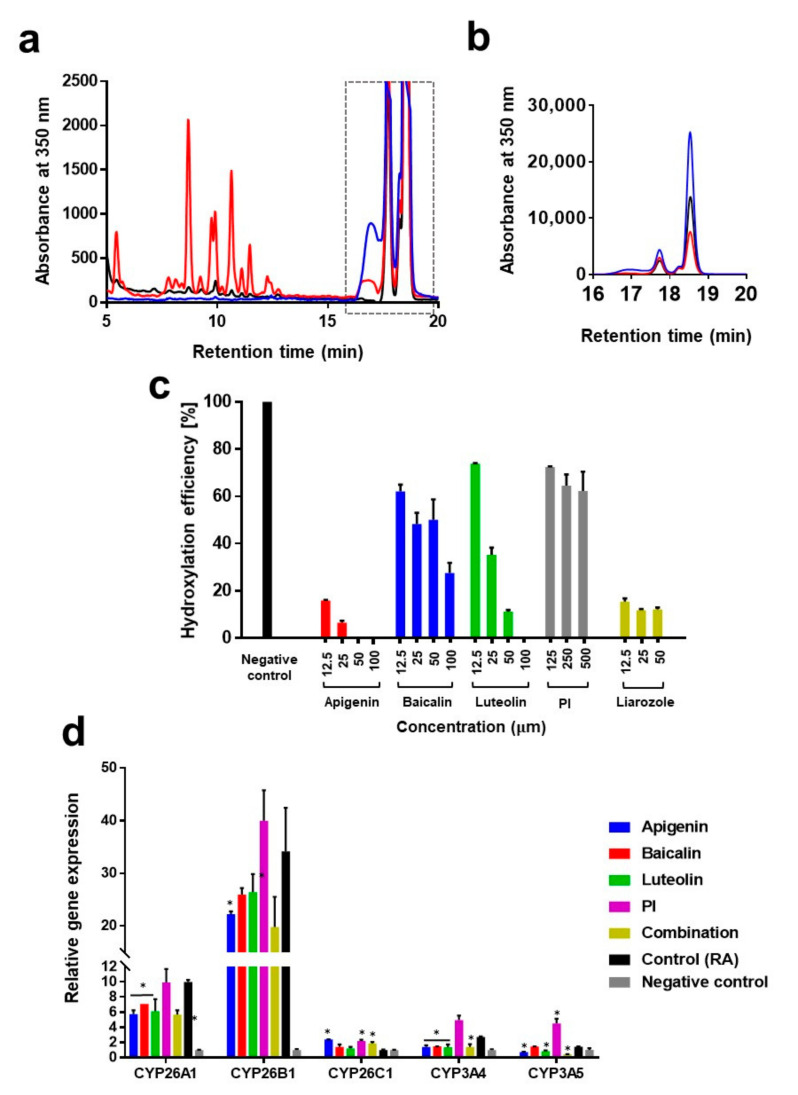
Screening RAMBA-mimicking compounds inhibiting RA hydroxylation. (**a**) HPLC spectrum for RA metabolites. HaCaT were treated with 1 µM RA for 48 h. Culture media and cell extracts were lyophilized and analyzed. For the inhibitor group (black line), 25 µM of apigenin was used. (**b**) An enlarged detail of the spectrum surrounded by the dotted box in (**a**). (**c**) RA hydroxylation efficiency, calculated as follows: 100 × peak area (oxidized RA metabolites, 5~15 min)/peak area (oxidized RA metabolites + RA). PI refers to prolinamidoethyl imidazole. (**d**) Relative mRNA expression of CYPs. HaCaT was cultured with candidates (25 µM) and RA for 48 h. Combination refers to co-treatment of apigenin, baicalin, and luteolin (each 25 µM). The bars denote the standard deviations. * Significantly different results (Student’s *t*-test, *p* < 0.05).

In the present study, we attempted to identify new RAMBA-mimicking materials, which are generally regarded as safe and non-irritant to the skin. RA and its metabolites metabolized by keratinocyte (HaCaT) were analyzed using HPLC, as described in previous studies [19]. Polar metabolites were observed at a retention time ranging from 5 to 15 min, and their production was found to be inhibited by inhibitors (Figure 2a). Furthermore, total RA (17~19 min) was found to decrease less when hydroxylation was inhibited, which implies that less exogenous RA was metabolized (Figure 2b). We had tested various substances and observed that most of them were not effective to inhibit RA hydroxylation while some candidates showed a significant level of inhibition. Figure 2c shows experimental results in which apigenin, baicalin, luteolin, and prolinamidoethyl imidazole effectively inhibited the hydroxylation or metabolism of retinoic acid by keratinocytes. Among these phytochemicals, apigenin showed a hydroxylation efficiency of 15.97% and 6.52% at concentrations of 12.5 and 25 µM, respectively, which is as potent as a positive control liarozole. Interestingly, one imidazole compound, prolinamidoethyl imidazole, which is widely used as an antioxidant in cosmetic products, showed a hydroxylation efficiency of approximately 60%; in other words, a potency of 45% compared to liarozole. Furthermore, not all types of imidazole were found to effectively inhibit the hydroxylation of RA, such as imidazole or phenyl benzimidazole sulfonic acid. Significant cytotoxicity of candidates was not observed (Supplementary Figure S2).

Next, we investigated whether RAMBA-mimicking materials could inhibit the hydroxylation of retinoic acid either directly or indirectly by regulating the expression of CYP enzymes. The expression of CYP enzymes in keratinocytes after treatment with retinoic acid (1 µM) were analyzed using RT-qPCR (Figure 2d). As expected, various CYP enzymes were expressed upon treatment with retinoic acid (1 µM). *CYP26A1* and *CYP26B1* were dominantly expressed, with 9.99- and 19.77-fold expression levels, respectively. This observation is in agreement with previous reports [26,30]. Furthemore, the expression of *CYP3A4* was found to be induced upon RA treatment, with a 2.77-fold expression. Although the precise roles and mechanisms of action were not elucidated in as much detail as CYP26, it seems that *CYP3A4* also contributes to the retinoids metabolism in the skin, considering that retinoids induce the expression of *CYP3A4* via a RXR/VDR-mediated pathway [31], in addition to recent reports on the expression and enzyme activity of CYP3A4 in human skin tissue [32]. Three phytochemicals, namely apigenin, baicalin, and luteolin, were found to inhibit the RA-induced overexpression of CYPs, with an inhibition efficacy of 40%. Interestingly, prolinamidoethyl imidazole did not affect the expression of CYPs, which implies that it inhibits RA hydroxylation by directly inhibiting CYPs, similar to azolic RAMBA.

#### 3.2.2. Enhancement of Pharmacological Efficacy of RA Boosters In Vitro, Ex Vivo, and In Vivo

**Figure 3:**
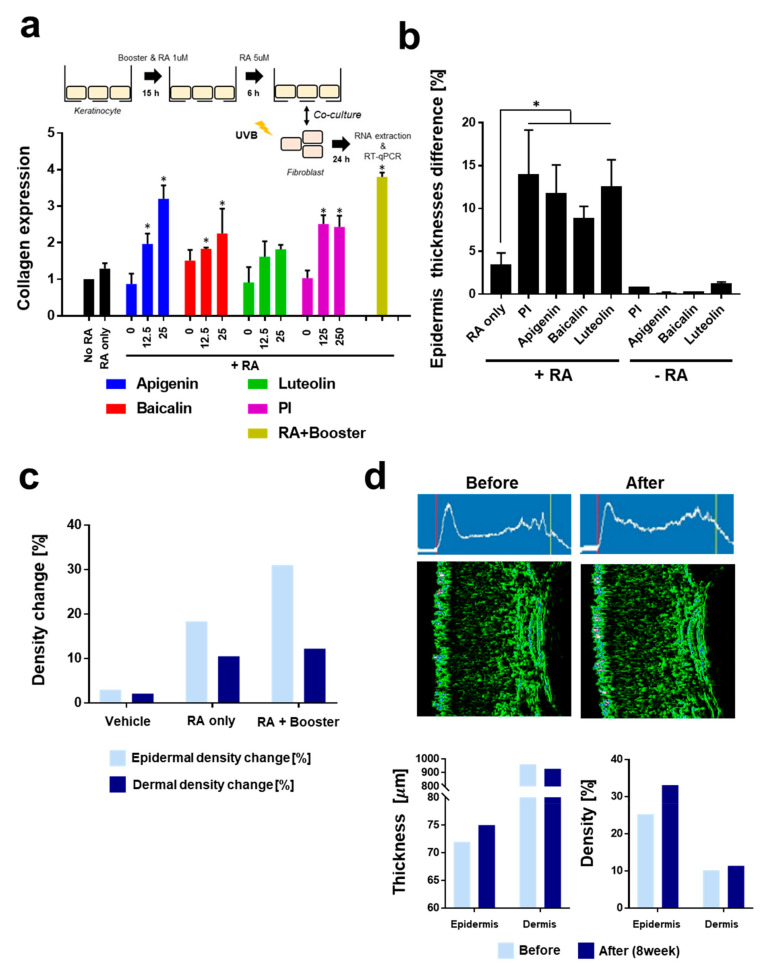
Efficacy of RA hydroxylation inhibitors in vitro, ex vivo, and in vivo. (**a**) Expression of collagen by fibroblasts co-cultured with keratinocytes. The *COL1A1* mRNA levels were analyzed by RT-qPCR. Keratinocytes (HaCaT) were cultured with RA or hydroxylation inhibitors prior to co-culture. A detailed experimental procedure is shown. Booster included 12.5 µM apigenin and baicalin and 125 µM prolinamidoethyl imidazole. (**b**) Epidermis thickening effects of RA and hydroxylation inhibitors in ex vivo experiments. An artificially reconstituted 3D skin model was used. (**c**) Change in the density of the epidermis and dermis in vivo. In a small clinical pilot study, O/W-type cream supplemented with RA (0.01%) and hydroxylation inhibitors (0.01% of apigenin, baicalin, and prolinamidoethyl imidazole) were applied onto the skin on the forearm of subjects twice a day for eight weeks. (**d**) Sonography analysis of RA- and booster-treated areas. Standard deviation bars are shown. * Significantly different results (Student’s *t*-test, *p* < 0.05).

We investigated how RA hydroxylation inhibitors may enhance the pharmacological efficacy of RA in vitro and ex vivo. Previous studies have shown that retinoic acid could ameliorate the photoaging and clinical signs. Retinoic acid has been shown to protect against the UV-induced loss of collagen [33,34]. To investigate the effect of RA hydroxylation by keratinocytes on the production of collagen by UV-treated fibroblasts, we newly designed the co-culture experiment as described in Figure 3a. Briefly, RA hydroxylase was induced by a small amount of RA, RA was added to the keratinocyte cultures, and fibroblasts were co-cultured with keratinocytes. Co-culture with keratinocytes decreased the expression of collagen by UV-treated fibroblasts by 1.29-fold, while collagen expression of fibroblasts without co-culture was 2.23 ± 0.13-fold. This implies that keratinocytes significantly hinder the pharmacological efficacy of retinoids in in vivo skin systems, as reported in a previous study [26]. Apigenin, baicalin, luteolin, and prolinamidoethyl imidazole were found to greatly enhance the collagen expression of fibroblasts in a dose-dependent manner. Among the phytochemicals, apigenin was the most potent, which is coincident with the results of the HPLC experiments (Figure 2a). The combination of 12.5 µM apigenin and baicalin and 125 µM prolinamidoethyl imidazole showed a higher collagen expression than each one alone. This implies that the inhibition of RA hydroxylation in both direct inhibition (prolinamidoethyl imidazole) and indirect inhibition, which modulates CYP enzyme expression (e.g., apigenin and baicalin), may be a more efficient strategy for enhancing the pharmacological efficacy of retinoids.

Next, we investigated the efficacy of RA on the epidermis in an artificially reconstituted 3D skin model (Figure 3b, Supplementary Figure S3). While treatment with RA or hydroxylation inhibitors alone did not result in a significant effect on epidermis thickening, co-treatment with RA and inhibitors significantly enhanced epidermis thickening.

In a small in vivo pilot study, we analyzed the skin of individuals subjected to different retinoids treatments using ultrasound sonography. Briefly, RA (0.01%) and retinoids boosters (apigenin, baicalin, and prolinamidoethyl imidazole, each 0.01%) were prepared in the O/W-type cream described in the method part. The final creams were applied on both sides of the forearm of a volunteer for two months. Interestingly, the density of the epidermis and dermis was found to increase after RA application, while application with a booster enhanced the efficacy of RA, increasing the density of the epidermis and dermis by 31.00% and 12.21%, respectively (Figure 3c). Compared to the dermis, the RA boosters were found to be more effective in epidermis thickening, showing an increase of 68.93%.

Figure 3d shows a detailed analysis of the sonography images for the region where the RA and boosters were applied. Similar to the density data, the thickness of epidermis was found to increase by about 3 µm (4.16%), while that of the dermis was almost unchanged. These results are similar to those reported in a previous study, in which retinaldehyde was found to increase epidermal thickness, while dermal thickness was unchanged, in a human clinical test [35].

Interestingly, an enhanced sonography echo signal (white line, Figure 3d) was observed in the transition region between the epidermis and dermis, also known as a subepidermal low-echogenic band (SLEB). Although little is known about SLEB and its structural basis, it has been suggested that elastosis or changes in collagen structure are responsible for an echolucent region [36]. Previous studies have also demonstrated that SLEB is related to photoaging—namely, the lower the SLEB echogenicity, the higher the rate of photoaging [36,37]. The findings presented in this study suggest that RA decreases the area of SLEB, which indicates that variations in SLEB may be an indicator of retinoids efficacy.

### 3.3. Enhancement of Retinol Efficacy Ex Vivo and In Vivo by Retinol Boosters (Pilot Study)

Compared to retinoic acid (tretinoin), retinol is considered to be more widely used for the improvement of skin appearance or as a remedy for photoaging skin due to its lower degree of irritancy and comparable efficacy. This has led the approval of retinol as a GRAS cosmetic ingredient by the FDA (CFR 21). In previous sections, we introduced the two strategies, boosting retinol-induced RAR activity and the inhibition of RA hydroxylation, separately. Here, we investigated how the combination of these two strategies could increase the pharmacological efficacy of retinol using both ex vivo and in vivo experiments. The retinoid booster investigated was comprised of camphor, apigenin, baicalin, and prolinamidoethyl imidazole.

**Figure 4:**
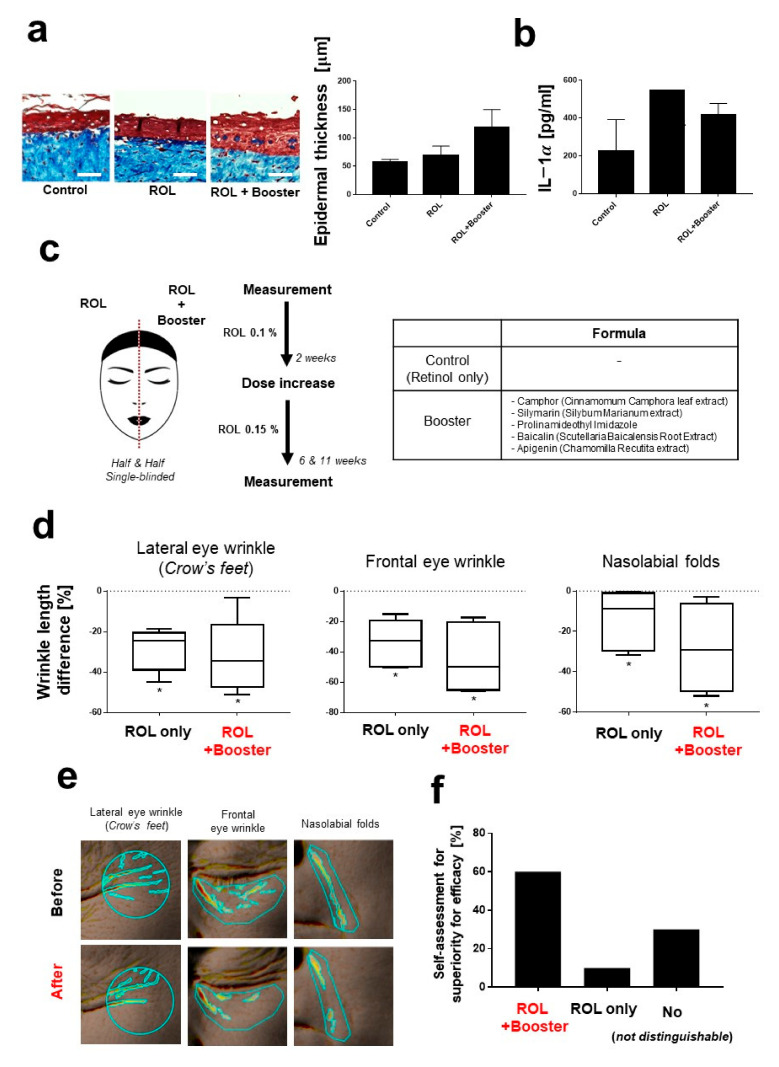
Efficacy of retinoids booster ex vivo and in vivo. (**a**) Epidermis thickening effects of retinol and retinol/booster. Scale bar, 50 µm. (**b**) Expression of IL-1α measured by ELISA. (**c**) Study design. Half & half, and single-blinded clinical test was performed. The table on the right shows the booster formulas for retinol. (**d**,**e**) Improvement in the appearance of wrinkles by retinol and retinol/booster (after 11 weeks of application). Three types of wrinkles were measured by Antera 3D. * Significantly different results (Paired *t*-test, *p* < 0.05). (**f**) Self-assessment for efficacy. Human subjects were asked which cream they thought was better at improving the appearance of their skin.

The ex vivo results for the 3D skin model are shown in Figure 4a. In this experiment, the retinol and retinol/booster combination treatment was topically applied onto the 3D skin model twice a week. Then, the 3D skin was sectioned and stained with Masson-trichrome staining. Treatment with retinol alone and with the retinol/booster combination resulted in an increase of the epidermis thickness of 20.83% and 104%, respectively. Retinol treatment did not show any significant difference compared to the non-treated control group, in agreement with a previous study [28]. By contrast, the retinol/booster combination treatment group showed a significant difference compared with the control. In addition, the inflammatory factor IL-1α, which extensively governs inflammation reactions in the skin, was also analyzed (Figure 4b) [38]. Previous studies have shown that retinoid induces diverse proinflammatory cytokines, including IL-1α, which may contribute to the retinoid-induced inflammation or irritation of the skin [17]. Treatment with retinol and retinol/booster did not significantly increase the excretion of IL-1α.

Next, we investigated how the retinol boosters enhance the pharmacological efficacy of retinol in terms of an improvement in the appearance of wrinkles in vivo. As shown in Figure 4c, two types of cream (a retinol-only cream and a retinol cream supplemented with boosters) were used to treat human subjects. The formulas of the creams are summarized in the table in Figure 4c.

As expected, treatment with retinol for 11 weeks noticeably improved the appearance of three types of wrinkles: lateral eye wrinkle (Crow’s feet), frontal eye wrinkle, and nasolabial folds (Figure 4d,e). Although it is difficult to directly compare our findings on the pharmacological efficacy of retinol to those reported in previous studies due to the use of different analysis tools and indexes (e.g., wrinkle depth, length, and area), the 20% improvement in the appearance of wrinkles observed in the present study is in agreement with those reported in the literature [39,40,41]. Interestingly, booster treatment was found to enhance the anti-wrinkle effect of retinol by 29.49%, 11.05%, and 103.04% for lateral eye wrinkle (Crow’s feet), frontal eye wrinkle, and nasolabial folds, respectively. Treatment with the retinol/booster combination greatly improved the appearance of nasolabial folds in terms of their length, which is generally considered to be a challenging aspect of this type of wrinkle, although the effect was not as marked as those observed after hyaluronic acid or calcium hydroxylapatite-based filler treatments [42,43]. Retinoids histologically increase skin elasticity by increasing collagen production, as mentioned in previous studies, which may have contributed to the amelioration in the appearance of nasolabial folds. In a single-blinded test, the majority of subjects reported that the retinol/booster combination was more effective in improving skin appearance than the retinol-only cream (Figure 4f).

Next, whether the booster could enhance the efficacy of other type of retinoids, such as retinoic acid, was also investigated. Treatment with retinoic acid, which is more pharmacologically potent than retinol, showed a greater improvement in the appearance of wrinkles than retinol (treatment for six weeks) (Supplementary Figure S4). Furthermore, treatment with the RA booster was found to enhance the pharmacological effects of RA on the appearance of wrinkles by 67.95%, 66.97%, and 76.90% for the three types of wrinkles (Supplementary Figure S4). It is worth noting that these clinical data were obtained from a pilot study with a limited number of subjects and it would be worthwhile to observe how human subjects respond to retinoids boosters in real-world applications. It was observed that co-treatment of retinol and booster resulted in higher statistical significance between baseline (0 day) and 12 weeks, especially for frontal eye wrinkles and nasolabial folds (0.037 (Retinol) vs. 0.030 (Retinol/Booster); 0.042 (Retinol) vs. 0.031 (Retinol/Booster), paired-test). The statistically significant differences between retinol and retinol/booster were not observed, which was thought to be due to a limited number of human subjects. Thus, large-scale clinical studies should be conducted in the future.

These observations implied that our comprehensive strategy could be applicable to not only retinol and tretinoin, but also to other retinoids, such as retinal, retinyl palmitate, and retinyl propionate, as well as other generation retinoids, including etretinate, adapalene, and trifarotene. Previous studies have shown that most retinoids induce therapeutic efficacy through RARs or RXRs [44]. For the high-generation retinoids, it is worth noting that adapalene is metabolized through either O-demethylation or hydroxylation, or combinations of these processes, although the metabolism and excretion of high-generation retinoids have not yet been clearly characterized (FDA NDA report (22–320); Study PK 91,005 (DCa/JF/92–020), Study RDS.03.SRE.4518). Taking this into account, the boosting of RAR activation by camphor and the inhibition of hydroxylation seems to enhance the pharmacological efficacy of a wide range of retinoids and their derivatives.

Moreover, some points deserve further consideration. Many research papers and clinical trials have demonstrated that the long-term use of retinoids increases tolerance to retinoid-induced irritation [45]. However, there is room for doubt whether this development of “tolerance to irritation” (also general termed “adaptation to irritation”) is due to a biological negative feedback mechanism for the homeostasis of retinoids in the body. It has already been proven that retinoids induce a negative feedback for retinoid homeostasis in several ways, such as in the retinol-induced overexpression of LRAT, which reduces the intracellular levels of biological active retinoids and retinoids efficacy [46,47], and the overexpression of CYPs highly related to RA resistance in the bone marrow [48]. Furthermore, it has not been clearly demonstrated whether the topical application of retinoids results in this tolerance or whether tolerance to retinoid-induced irritation is correlated to pharmacological efficacy. If tolerance truly exists and can hinder the long-term therapeutic efficacy of retinoids, our approach could overcome this issue and result in both a higher efficacy and a higher patient compliance, not only for a cosmetic use, but also for dermatologic therapy.

In addition, it is worth considering why individuals experience different therapeutic efficacies towards retinoids. As reported in previous studies, a wide range of responses to retinoids have been observed in humans. Several studies have shown that genetic variations at the single nucleotide polymorphism (SNP)-level could affect the concentration of retinoids in the body [49], even under pathogenic conditions. For example, it has been shown that polymorphisms in CYP26B1 may elevate the level of retinoic acid and increase the risk of Crohn’s disease [50]. Similarly, polymorphism in RARA could affect clinical resistance to retinoic acid [51]. In another study, researchers found that genetic variations in CYP26s resulted in different retinoic acid metabolism [52]. Although it has not been clearly verified whether the efficacy of retinoids on photoaging is associated with genetic variations, the association of genetic variations with therapeutic efficacy is likely to exist, as suggested in previous studies. As highlight in cases of APL, different responses to retinoids due to genetic variations may be key to understanding this difference. If this is demonstrated in the future, it may possible to predict individual responses to topically applied retinoids, and thus offer a genetically personalized or customized strategy to maximize the therapeutic efficacy. In this respect, our research may contribute to this by providing a basis for the development of customized booster formulas.

Herein, the enhancement of the therapeutic efficacy of retinoids was demonstrated through the use of boosters in a small scale pilot study. This strategy could contribute to increase the use of retinoids in a wide range of applications, not only for cosmetic use, but also in therapeutic use for the treatment of dermatological diseases.

### 3.4. Case study: Long-Term Use of Retinol and Retinol/Booster for 200 Days

Among the subjects who participated in the pilot study for retinol use, some subjects (six women) consented to use retinol and retinol/booster for an extended period of time. This allowed us to follow up on how the long-term use of retinol and boosters can improve the appearance of wrinkles in a small case study. Despite its small size, this case study has the potential to provide meaningful insights on the long-term use of retinol (>6 months), which has been less extensively researched than retinoic acid. To the best our knowledge, a limited number of studies have been performed on the long-term (>6 months) [53], with most studies on the therapeutic efficacy of retinol for photoaging (or anti-wrinkle properties) being performed over a 3- to 12-week period [7,54,55].

**Figure 5:**
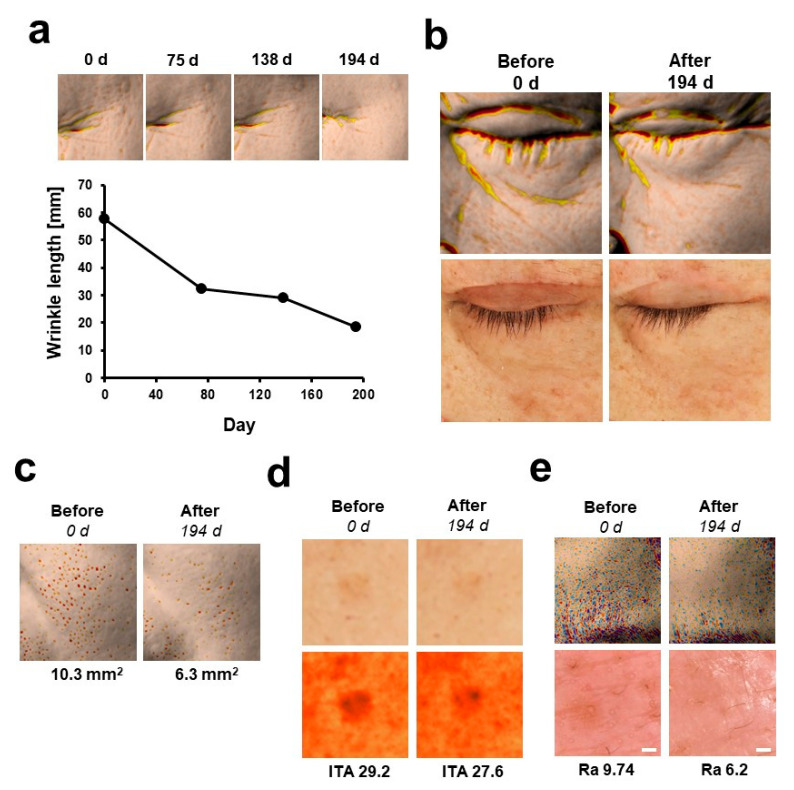
Long-term use of retinol and booster (194 days). Data for the most responsive human subject for each clinical features are shown. All data were analyzed by Antera 3D. (**a**) Improvement in the appearance of crow’s feet. (**b**) Improvement in the appearance of frontal eye wrinkle. (**c**) Improvement in the appearance of pores (front cheek area). (**d**) Improvement in hyperpigmentation spots. (**e**) Improvement in skin texture. Skin surface (lower row) was evaluated by microscopy. Scale bar (white), 200 µm.

As shown in Figure 5a, significant improvements in the appearance of wrinkles were observed in the subjects, which were apparent to the naked eye. In fact, one subject showed an improvement of 68% for crow’s feet. Focusing on this specific type of winkle, co-treatment with retinol and boosters was found to exhibit a more rapid improvement in wrinkle appearance than retinol alone, implying a shorter onset-time (Supplementary Figure S5). A marked improvement was also observed in the case of frontal eye wrinkles, as shown in Figure 5b.

In addition to the different wrinkle types, marked improvements were also observed in dilated pores, hyperpigmentation, and the texture of skin, which coincides with the results of previous studies (Figure 5c–e) [40]. The improvement in the appearance of dilated pores after retinol application seems to be have been achieved by the normalization of keratinocyte keratinization and sebum production [56], as well as by an enhancement of the elasticity and tensile strength of the skin, which are negatively correlated with pore size and density [57]. In the case of hyperpigmentation, a decrease of ITA (1.6, Individual Typology Angle) was observed. When the skin texture was analyzed using Antera 3D, one particular indicator of skin texture, namely roughness (Ra), was found to decrease from 9.74 to 6.2 (36% decrease). The qualitative analysis of skin on the forehead of the subjects using microscopy also demonstrated a reduction in the appearance of fine wrinkles and pores and an improvement in the overall evenness of the skin surface (Figure 5e). Interestingly, retinol treatment also resulted in a greater reflectance of light off of the skin, also known as “skin radiance”, although the characterization and quantification of this characteristic of the skin remain controversial and have yet to be established [58,59]. When measuring the effect of retinol on these areas, treatment appeared to increase light reflectance by 2.27-fold. However, further study and a standardized method for the measurement of skin radiance will be needed to verify these findings.

## 4. Conclusions

Retinoids are widely used for a wide range of purposes, including in cosmetic use to improve the appearance of wrinkles and as a remedy for skin diseases. Among the different types of retinoids, retinoic acid and retinol (a reduced form of retinoic acid) have been frequently used and studied for their efficacy on photoaging. However, humans possess several metabolic mechanisms against high physiological levels of exogenous retinoid, resulting in an innate mechanism for the maintenance of retinoid homeostasis in tissues. Furthermore, retinoids are known to cause skin irritation in many users. As a result, a higher pharmacological efficacy at a lower dosage is needed to improve patient compliance.

In this study, we attempted to enhance the therapeutic efficacy of retinoids for cosmetic use using two strategies: (1) enhancing retinoid-induced RAR activation, and (2) inhibiting the hydroxylation of retinoic acid. We demonstrated that camphor enhanced retinol-induced RAR-γ activation, as well as the therapeutic efficacies of retinol in vitro and ex vivo. In terms of the inhibition of RA hydroxylation in skin tissue, prolinamidoethyl imidazole and several phytochemicals (apigenin, baicalin, and luteolin) were found to inhibit the hydroxylation of retinoic acid mediated by keratinocyte. In vitro and ex vivo experiments also showed that these hydroxylation inhibitors could enhance the therapeutic efficacy of retinoic acid.

In addition, the results of a pilot study to investigate the efficacy of retinoids in terms of improving the appearance of wrinkles and their action on photoaging showed that boosters enhanced the efficacy of retinoids. By developing a strategy to achieve a higher efficacy of retinoids with a lower dosage, this study contributes to improving the patient compliance and consumer benefits of retinoids not only for cosmetic use, but also for their medical use. As a result, this study contributes to our understanding of the mechanism underlying the action of retinoids in general and provides a basis for the development of retinoids-based products in the future.

## Data Availability

The data that support the findings of this study are available from the corresponding author on request.

## Supplementary Materials

The following supporting information can be downloaded at: https://www.mdpi.com/article/10.3390/pharmaceutics14112412/s1, Figure S1: Cell viability for camphor treatment. Figure S2: Cell viability for RA hydroxylation inhibitors treatment. Figure S3: Epidermis thickening effects of RA and hydroxylation inhibitors in ex vivo experiments. Figure S4: In vivo study for retinoids and retinoids/booster. Figure S5: Long-term use of retinol/booster for 194 days.
