# Supplementary material for: Enhancement of Efficacy of Retinoids through Enhancing Retinoid-Induced RAR Activity and Inhibiting Hydroxylation of Retinoic Acid, and Its Clinical Efficacy on Photo-Aging
Source: Pharmaceutics. 2022 Nov 8;14(11):2412. doi: 10.3390/pharmaceutics14112412 (PMC9692645; doi:10.3390/pharmaceutics14112412)
Supplement: Supplementary file 1 [file pharmaceutics-14-02412-s001.zip › pharmaceutics-1985027-supplementary.pdf]

pharmaceutics

MDPI

Supplementary Materials

Enhancement of Efficacy of Retinoids through Enhancing Retinoid-Induced RAR Activity and Inhibiting Hydroxylation of Retinoic Acid, and Its Clinical Efficacy on Photo-Aging

Seongsu Kang, Hyejin Lee, Seung-Hyun Jun *, Sun-Gyoo Park and Nae-Gyu Kang *

LG Household and Health Care R&D Center, Seoul 07795, Korea
* Correspondence: junsh@lghnh.com (S.-H.J.); ngkang@lghnh.com (N.-G.K.); Tel.: +82-2-6980-1239 (S.-H.J.); +82-2-6980-1533 (N.-G.K.)

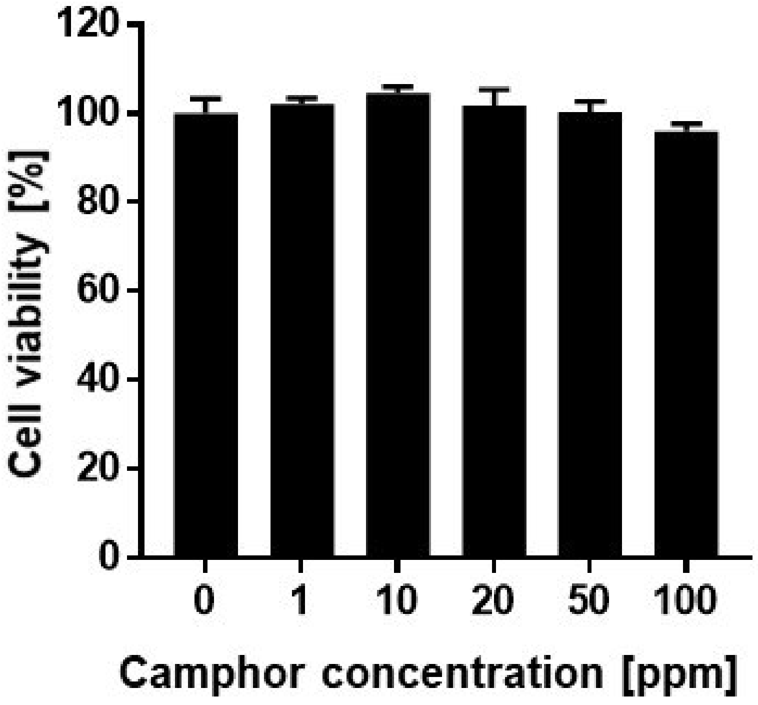

Supplementary Figure S1. Cell viability for camphor treatment. CCK-8 (Cell Counting Kit-8, Dojindo, Rockville, Maryland, USA) was performed.

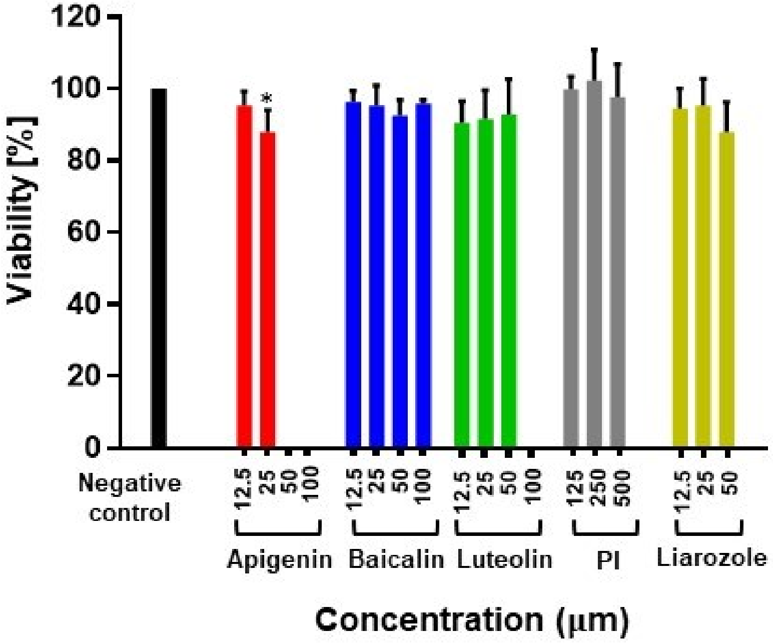

Supplementary Figure S2. Cell viability for RA hydroxylation inhibitors treatment. CCK-8 (Cell Counting Kit-8, Dojindo, Rockville, Maryland, USA) was performed. The.

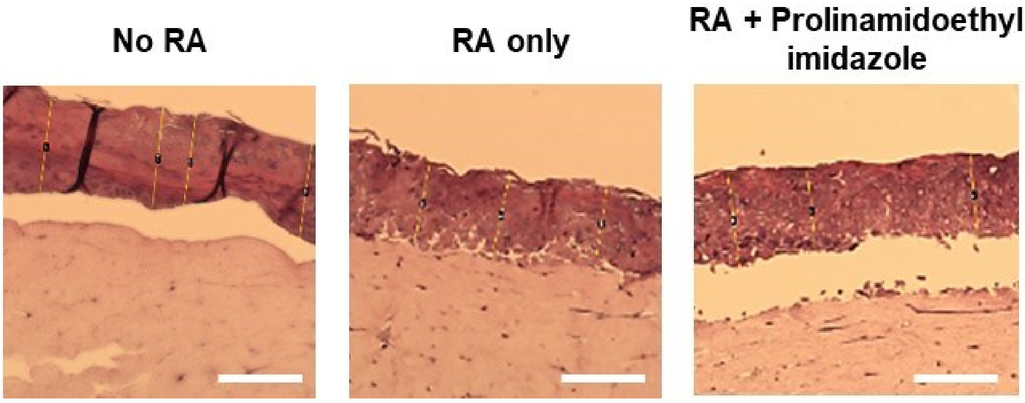

Supplementary Figure S3. Epidermis thickening effects of RA and hydroxylation inhibitors in *ex-vivo* experiments. Artificially reconstituted of 3D skin model was used. The tissue was stained by hematoxylin-eosin (H&E). Scale bar, 50 μm.

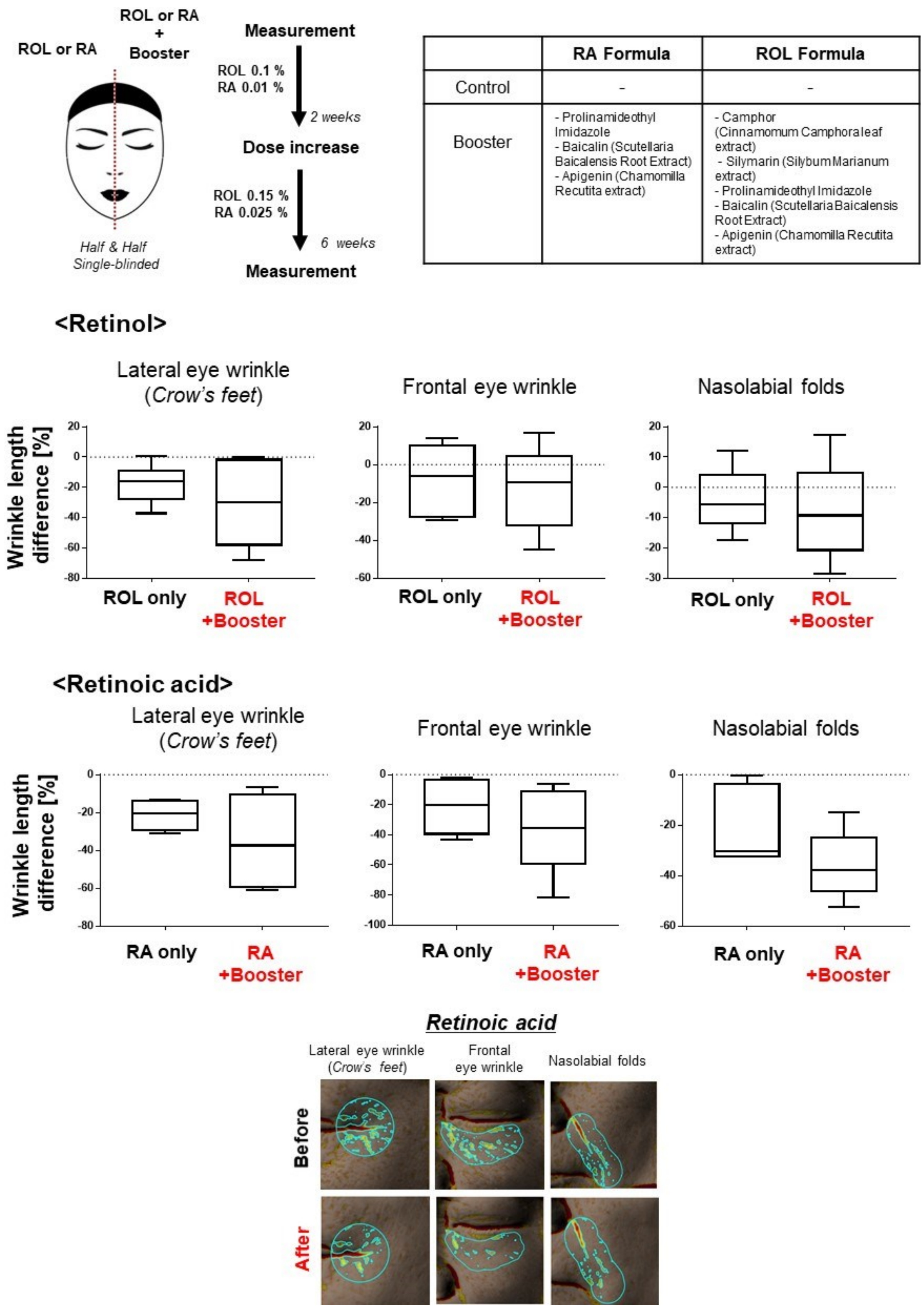

Supplementary Figure S4. *In-vivo* study for retinoids and retinoids/booster. Half & half, and single-blinded clinical test was performed for 6 weeks. Three types of wrinkles were measured by Antera 3D.

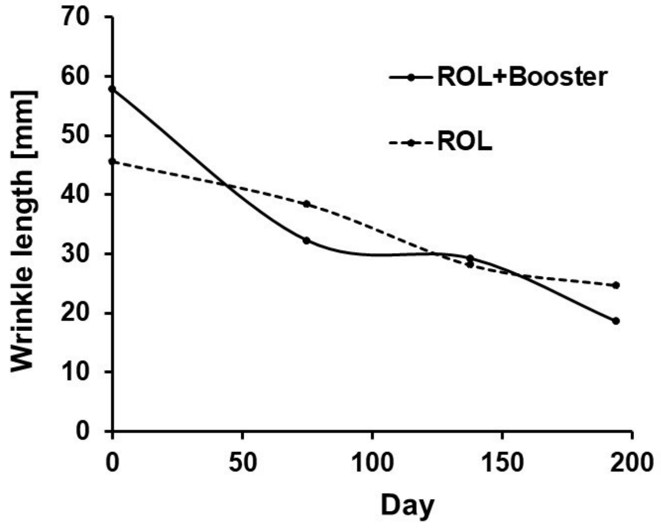

Supplementary Figure S5. Long-term use of retinol/booster for 194 days. Improvement of crow's feet was analyzed. Data for the most responsive human subject was shown. All data was analyzed by Antera 3D.
